# Effects of 3-Month Exposure to E-Cigarette Aerosols on Glutamatergic Receptors and Transporters in Mesolimbic Brain Regions of Female C57BL/6 Mice

**DOI:** 10.3390/toxics8040095

**Published:** 2020-10-29

**Authors:** Hasan Alhaddad, Woonyen Wong, Adam T. Sari, Laura E. Crotty Alexander, Youssef Sari

**Affiliations:** 1 Department of Pharmacology and Experimental Therapeutics, College of Pharmacy and Pharmaceutical Sciences, the University of Toledo, Toledo, OH 43614, USA; Hasan.Alhaddad@rockets.utoledo.edu (H.A.); Woonyen.Wong@rockets.utoledo.edu (W.W.); Adam.Sari@rockets.utoledo.edu (A.T.S.); 2 Pulmonary and Critical Care Section, VA San Diego Healthcare System, 3350 La Jolla Village Dr, MC 111J, San Diego, CA 92161, USA; lcrotty@ucsd.edu; 3 Department of Medicine, Division of Pulmonary and Critical Care, University of California at San Diego (UCSD), La Jolla, CA 92093, USA

**Keywords:** e-cigarettes, nicotine, JUUL, mGluR1, mGluR5, GLT-1, xCT, nucleus accumbens, hippocampus

## Abstract

Electronic cigarettes (e-cigs) use has been dramatically increased recently, especially among youths. Previous studies from our laboratory showed that chronic exposure to e-cigs, containing 24 mg/mL nicotine, was associated with dysregulation of glutamate transporters and neurotransmitter levels in the brain of a mouse model. In this study, we evaluated the effect of three months’ continuous exposure to e-cig vapor (JUUL pods), containing a high nicotine concentration, on the expression of glutamate receptors and transporters in drug reward brain regions such as the nucleus accumbens (NAc) core (NAc-core), NAc shell (NAc-shell) and hippocampus (HIP) in female C57BL/6 mice. Three months’ exposure to mint- or mango-flavored JUUL (containing 5% nicotine, 59 mg/mL) induced upregulation of metabotropic glutamate receptor 1 (mGluR1) and postsynaptic density protein 95 (phosphorylated and total PSD95) expression, and downregulation of mGluR5 and glutamate transporter 1 (GLT-1) in the NAc-shell. In addition, three months’ exposure to JUUL was associated with upregulation of mGluR5 and GLT-1 expression in the HIP. These findings demonstrated that three-month exposure to e-cig vapor containing high nicotine concentrations induced differential effects on the glutamatergic system in the NAc and HIP, suggesting dysregulation of glutamatergic system activity in mesolimbic brain regions.

## 1. Introduction

Over recent years, electronic cigarettes (e-cigs) use has been dramatically increased, especially among youths [1,2]. Despite the application of e-cigs as a smoking cessation tool, its use was associated with different detrimental effects such as DNA damage [3] and decreased host defenses [4]. The use of e-cigs, such as JUUL pods containing 5% nicotine, has been increased recently compared to older e-cig devices, especially among the young population [5,6]. There is a myriad of JUUL pods introduced to the market with improved palatability and higher concentration of nicotine (5%) content, which trigger the description of JUUL use as an epidemic by public health agencies (e.g. FDA) [7,8]. Importantly, the uses of e-cigs induce a comparable urge to smoke and increase the desire for conventional combustible smoking [9]. Studies have demonstrated an addictive-like behavior with e-cigs use in clinics [10]. Nicotine represents the addictive component in e-cigs, which is found in most e-liquids in the market [2,11]. The effects of tobacco and e-cigs smoking are sex-dependent. In humans, nicotine may induce differential effects in females than males (for review see [12]), and nicotine replacement therapies were found to be less effective in females compared to males [13,14]. Studies revealed that females are more sensitive than males and exhibited higher reinforcement toward e-cigs use [15]. It has also been revealed that 38% of female vs. 27% of male current smokers tried to smoke e-cigs as of 2013 in the US [16]. These data suggested that females may have a greater tendency to use e-cigs than males.

Recent studies from our laboratory showed that chronic exposure to e-cigs was associated with dysregulation of glutamate transporters and neurotransmitters levels in the brain [17,18]. Exposure to e-cigs for six months induced downregulation of glutamate transporter 1 (GLT-1) in the striatum, and downregulation of cystine/glutamate antiporter (xCT) in the striatum and hippocampus (HIP) of female CD-1 mice [17]. It is important to note that GLT-1 is responsible for the clearance of the majority of extracellular synaptic glutamate [19,20]. Alternatively, chronic e-cig exposure in male C57BL/6 mice was associated with a reduction in dopamine concentration and increase in glutamate concentration in the striatum, while GABA concentration was decreased in the frontal cortex [18]. These studies suggest that chronic e-cig exposure alters glutamate homeostasis. It is noteworthy that chronic exposure to drugs of abuse (e.g., nicotine, ethanol, methamphetamine, hydrocodone and cocaine) was associated with downregulation of GLT-1 [21,22,23,24] and xCT protein expression [23,24,25] in key reward brain regions in P rats. In these studies, β lactams, known to upregulate GLT-1 and xCT expression, were tested and showed attenuating effects on drug-seeking behaviors.

To the best of our knowledge, less is known about the effect of exposure to e-cigs (JUUL pods containing 5% nicotine) on the expression of metabotropic glutamate receptors. Group 1 metabotropic glutamate receptors (mGluR1 and mGluR5) are mainly located on the post-synaptic region and are involved in drug dependence, development and maintenance (for review see [26,27]). These receptors have a pivotal role in mediating memory and learning [28], regulating ionotropic glutamate receptors, i.e., N-methyl-D-aspartate (NMDA) and a-amino-3-hydroxy-5-methyl-4-isoxazole propionic acid (AMPA) receptors [29], and synaptic plasticity [30]. This suggests that these receptors are major regulators of glutamate homeostasis. Studies have shown that mGluR5 is highly expressed in the nucleus accumbens (NAc) and HIP [31], and its activity is critical for mediating nicotine reinforcement [32,33,34]. mGluR5 antagonism attenuated nicotine seeking and self-administration [34,35]. Alternatively, mGluR1 mRNA and protein expression were increased after repeated exposure to nicotine in the ventral tegmental area (VTA) and amygdala in rats [36]. It is important to note that mGLUR1 expression is relatively lower in mesocorticolimbic brain regions as compared to mGluR5 (for review see [37]). Furthermore, the blockade of mGluR1 reduced the reinstatement to nicotine self-administration [38]. Exposure to e-cigs containing nicotine induced comparable plasma nicotine levels as compared to conventional tobacco smoking [39]. However, other additives in e-cigs might contribute to the addictive/harmful effects of e-cig vaping. For instance, there is an increase in the tendency to smoke menthol-, candy- or fruit-flavored e-cigs over tobacco-flavored e-cigs [40]. It has been shown that menthol-flavored e-cigs promote dependence and impact the smoking cessation outcomes (for review see [41]). We investigated in this study the effects of exposure to different flavored e-cigs’ vapor (JUUL pods), containing a high nicotine concentration, on mGluR1, mGluR5, GLT-1, and xCT expression in the NAc core (NAc-core), NAc shell (NAc-shell) and HIP, which represent key brain regions involved in drug reward behaviors [42,43].

## 2. Materials and Methods

### 2.1. JUUL Exposure

Female C57BL/6 mice at the age of 6–8 months were used in this study. We focused on female mice as it has been revealed previously that there is a sex-dependent effect of nicotine, and that female rats showed higher nicotine-seeking behaviors compared to males [44,45]. Mice were purchased from Envigo Inc. (Indianapolis, IN, USA), and placed in individual sections of a full-body exposure chamber, SciReq inExpose system (SCIREQ Emka Technologies Company, Montreal, QC, Canada) for 20 min daily for 5 days per week in a 3-month period. Two groups of mice were exposed to e-cig aerosols from either Mint JUUL pods (e-cig mint group, *n* = 5–6) or Mango JUUL pods (e-cig mango group, *n* = 5–6) containing 5% nicotinic salts (59 mg/mL). Another group of mice was exposed to room air only in an identical chamber for 20 min daily for 5 days per week, for 3 months (air control group, *n* = 5–6). The e-cigs were activated for 4 s followed by 16 s of room air at 2 L/s, using a negative pressure of 2 L/s. We created a 3D-printed adapter to fit the JUUL device as described previously in [46]. The last exposure was performed 30 min prior to animal euthanization. All animal experiments were approved by the Animal Care and Use Committee of the University of California San Diego (identification code: s16021; date of approval: 1 May 2014, amended/approvals every 6 months).

### 2.2. Brain Tissue Harvesting

**Figure 1:**
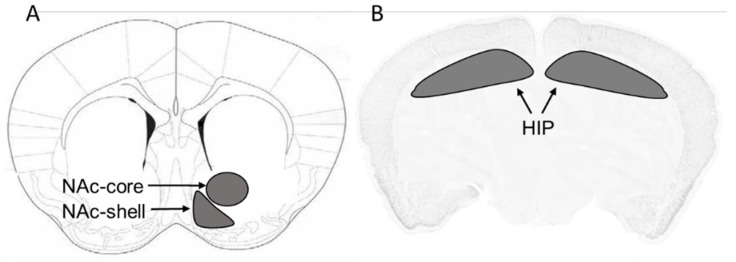
Schematic diagram showing the location of (**A**) the nucleus accumbens core (NAc-core) and shell (NAC-shell), bregma 1.42, and (**B**) hippocampus (HIP), bregma −2.46 mm, in mouse brain.

Mice were euthanized by CO_2_ inhalation 30 min after their last pods or air control exposure. Mice were rapidly decapitated with a guillotine and the brains were removed and stored at −80 °C. The NAc-core, NAc-shell and HIP were micropunched stereotaxically and isolated using a cryostat apparatus maintained at −20 °C. Following visualized landmarks, we isolated the brain regions of interest using The mouse brain in stereotaxic coordinates [47] as it is shown in Figure 1.

### 2.3. Western Blot Technique

mGluR1, mGluR5, GLT-1, xCT, phospho-postsynaptic density protein (*p*-PSD95), and PSD95 protein expression were measured in the NAc-core, NAc-shell and HIP using Western blot technique. Brain regions were homogenized using filtered lysis buffer (2.5 mL 1 M Tris HCL, 2.5 mL 3 M NaCl, 0.1 mL 0.5 M EDTA, 2.5 mL 10% NP-40, 5 mL 10% Triton, 0.5 mL 10%SDS and 36.9 mL Millipore water), which includes protease and phosphatase inhibitors. A protein assay (Bio-Rad, Hercules, CA, USA) was utilized to determine the amount of protein in each brain tissue sample. An equal amount of protein from each sample was then loaded into 10% polyacrylamide gels. Separated proteins in gels were transferred electrophoretically into PVDF membranes, which were further blocked with 5% fat-free milk in Tris-buffered saline, including Tween-20 (TBST). Membranes were then incubated at 4 °C (overnight) with primary antibodies: rabbit anti-GLT-1 (1:1000; Abcam, Inc., Branford, CT, USA), rabbit anti-xCT (1:1000; Abcam, Inc.), rabbit anti-mGluR1 (1:1000; Abcam, Inc.), rabbit anti-mGluR5 (1:1000; MilliporeSigma, Burlington, MA, USA), rabbit anti-phospho PSD95 (1:1000; Abcam, Inc.), rabbit anti-PSD95 (1:1000; Abcam Inc.) and the control loading protein mouse anti-β- tubulin (1:1000; BioLegend Inc., San Diego, CA, USA). The appropriate secondary antibodies (1:5000) were added to their respective membranes, after 5 washes with TBST, for 90 min at room temperature. Blot images were digitalized using the GeneSys imaging system after incubation of the membranes with chemiluminescent reagents, Super Signal West Pico (ThermoFisher Scientific, Waltham, MA, USA). Finally, quantifications of the expression of mGluR1, mGluR5, GLT-1, xCT and β-tubulin were performed using ImageJ software (developed by the National Institutes of Health, DC, USA). Air control group data were represented as 100% (relative to air control) to determine the changes in the expression of the target proteins, as illustrated in our previous study [23].

### 2.4. Statistical Analyses

GraphPad Prism v6.0 or v8.0 (Graphpad Holdings, LLC, CA, USA) was used to conduct the statistical analyses. Data obtained were analyzed by one-way ANOVA followed by the Newman–Keuls post hoc multiple comparison test to analyze the differences in the protein expression between mint- and mango-flavored JUUL pods-exposed groups relative to air controls. Data were analyzed as a percentage (relative to air control values) ratio to the loading control protein, β-tubulin. *p* value of ≤0.05 was considered statistically significant.

## 3. Results

### 3.1. Effect of Three-Month Exposure to JUUL on mGluR1 Protein Expression

**Figure 2:**
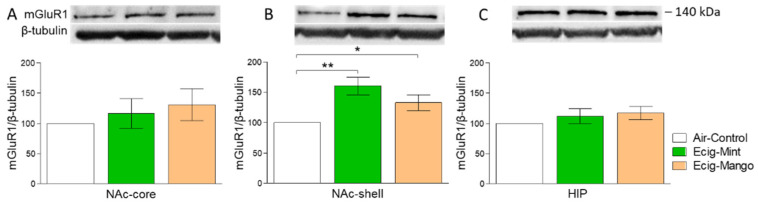
Effect of 3-month JUUL pods exposure on mGluR1 protein expression. (**A**) There was no significant change in the protein level of mGluR1 in the NAc-core. (**B**) mGluR1 expression was significantly upregulated in e-cig mint (*p* < 0.01) and e-cig mango (*p* < 0.05) groups as compared to the air control group in the NAc-shell. There was no significant difference between e-cig mint and e-cig mango groups. (**C**) No significant change in mGluR1 expression was found in the HIP. Data expressed as mean ± SEM; * *p* < 0.05 and ** *p* < 0.01; *n* = 5–6/group. Abbreviations: NAc-core, nucleus accumbens core; NAc-shell, nucleus accumbens shell; HIP, hippocampus.

Three-month exposure to mint- or mango-flavored JUUL containing 59 mg/mL nicotine for three months did not alter the expression of the mGluR1 level in the NAc-core compared to the air control as analyzed by one-way ANOVA followed by the Newman–Keuls post hoc test (F_2,14_ = 0.616, *p* > 0.05; *n* = 5–6/group, Figure 2A). However, three-month exposure to JUUL pods induced significant upregulation of mGluR1 expression in the NAc-shell of e-cig mint and e-cig mango groups (F_2,14_ = 7.35, *p* = 0.006; *n* = 5–6/group) as compared to the air control group. The Newman–Keuls post hoc test did not show any significant difference between e-cig mint and e-cig mango groups (Figure 2B). Like the NAc-core, three-month exposure to JUUL pods did not alter the expression of mGluR1 levels in the HIP (F_2,14_ = 0.989, *p* > 0.05; *n* = 5–6/group, Figure 2C).

### 3.2. Effect of Three-Month Exposure to JUUL on mGluR5 Protein Expression

**Figure 3:**
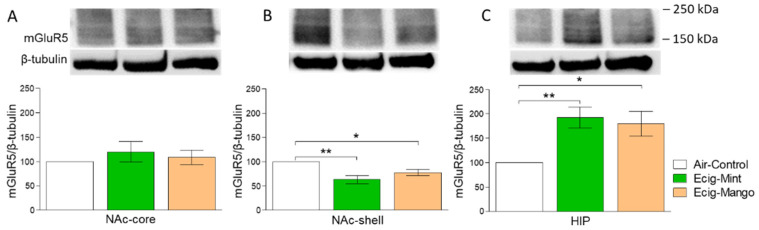
Effect of 3-month JUUL pods exposure on mGluR5 protein expression. (**A**) There was no significant change in the protein level of mGluR5 in the NAc-core. (**B**) mGluR5 expression was significantly downregulated in e-cig mint (*p* < 0.01) and e-cig mango (*p* < 0.05) groups as compared to the air control group in the NAc-shell. There was no significant difference between e-cig mint and e-cig mango groups. (**C**) mGluR5 expression was significantly upregulated in e-cig mint (*p* < 0.01) and e-cig mango (*p* < 0.05) groups as compared to the air control group in the HIP. There was no significant difference between e-cig mint and e-cig mango groups. Data expressed as mean ± SEM, * *p* < 0.05 and ** *p* < 0.01; *n* = 5–6/group. Abbreviations: NAc-core, nucleus accumbens core; NAc-shell, nucleus accumbens shell; HIP, hippocampus.

The effect of exposure to mint- or mango-flavored JUUL pods for three months on the expression of mGluR5 was also measured. There was no significant change in the expression of mGluR5 among e-cig mint, e-cig mango and air control groups in the NAc-core (F_2,14_ = 0.474, *p* > 0.05; *n* = 5–6/group, Figure 3A). One-way ANOVA followed by the Newman–Keuls post hoc test revealed that three-month exposure to JUUL pods was associated with significant downregulation of mGluR5 expression in e-cig mint (*p* < 0.01) and e-cig mango (*p* < 0.05) groups as compared to the air control group (F_2,14_ = 9.63, *p* = 0.002; *n* = 5–6/group) in the NAc-shell. However, there was no significant difference between e-cig mint and e-cig mango groups (Figure 3B). Additionally, three-month exposure to JUUL pods induced upregulation of mGluR5 expression in the HIP of e-cig mint (*p* < 0.01) and e-cig mango (*p* < 0.05) groups as compared to the air control group (F_2,14_ = 7.02, *p* = 0.007; *n* = 5–6/group). There was no significant difference between the e-cig mint and e-cig mango groups (Figure 3C).

### 3.3. Effect of Three-Month Exposure to JUUL on GLT-1 Protein Expression

**Figure 4:**
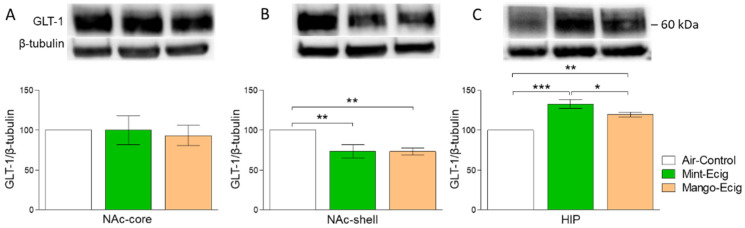
Effect of 3-month JUUL pods exposure on GLT-1 protein expression. (**A**) Statistical analysis did not show any significant change in GLT-1 protein level in the NAc-core. (**B**) GLT-1 expression was significantly increased in e-cig mint (*p* < 0.01) and e-cig mango (*p* < 0.01) groups as compared to the air control group in the NAc-shell. There was no significant difference in GLT-1 expression between e-cig mint and e-cig mango groups. (**C**) GLT-1 expression was significantly upregulated in e-cig mint (*p* < 0.0001) and e-cig mango (*p* < 0.001) groups as compared to the air control group in the HIP. However, the e-cig mint group showed significant upregulation in GLT-1 expression as compared to the e-cig mango group (*p* < 0.05). Data expressed as mean ± SEM; * *p* < 0.05, ** *p* < 0.01 and *** *p* < 0.001; *n* = 5–6/group. Abbreviations: NAc-core, nucleus accumbens core; NAc-shell, nucleus accumbens shell; HIP, hippocampus.

We further intended to measure the effect of exposure to mint- or mango-flavored JUUL pods for three months on GLT-1 expression. Data analysis did not reveal any significant difference in the expression of GLT-1 in the NAc-core among e-cig mint, e-cig mango and air control groups (F_2,14_ = 0.11, *p* > 0.05; *n* = 5–6/group, Figure 4A). One-way ANOVA followed by the Newman–Keuls post hoc test showed that JUUL exposure induced significant downregulation of GLT-1 expression in e-cig mint (*p* < 0.01) and e-cig mango (*p* < 0.01) groups as compared to the air control group in the NAc-shell (F_2,14_ = 10.18, *p* = 0.0019; *n* = 5–6/group). However, there was no significant difference between e-cig mint and e-cig mango groups (Figure 4B). In contrast to the NAc-shell, GLT-1 expression was upregulated in the HIP of e-cig mint (*p* < 0.001) and e-cig mango (*p* < 0.01) groups as compared to the air control group (F_2,12_ = 20.41, *p* = 0.0001; *n* = 5/group, Figure 4C). The Newman–Keuls post hoc test showed a significant increase in GLT-1 expression in the e-cig mint group as compared to the e-cig mango group (*p* < 0.05, Figure 4C).

### 3.4. Effect of Three-Month Exposure to JUUL Pods on xCT Protein Expression

**Figure 5:**
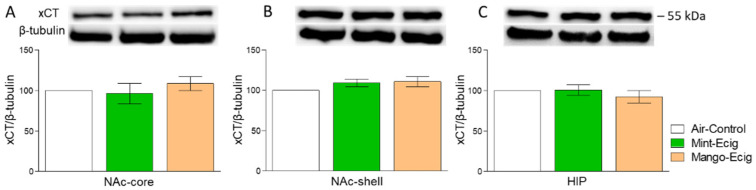
Effect of 3-month JUUL pods exposure on xCT protein expression. (**A**) There was no significant change in xCT expression between air control, e-cig mint and e-cig mango groups in the NAc-core. (**B**) xCT expression was not changed between all groups in the NAc-shell. (**C**) There was no significant change in xCT expression between all groups in the HIP. Data expressed as mean ± SEM; *n* = 5–6/group. Abbreviations: NAc-core, nucleus accumbens core; NAc-shell, nucleus accumbens shell; HIP, hippocampus.

Three-month exposure to JUUL pods did not alter xCT protein expression in all studied brain regions. Data analysis did not reveal any significant change in xCT expression among e-cig mint, e-cig mango and air control groups in the NAc-core (F_2,14_ = 0.57, *p* > 0.05; *n* = 5–6/group, Figure 5A), NAc-shell (F_2,14_ = 1.74, *p* > 0.05; *n* = 5–6/group, Figure 5B) and HIP (F_2,14_ = 0.66, *p* > 0.05; *n* = 5–6/group, Figure 5C).

### 3.5. Effect of Three-Month Exposure to JUUL Pods on p-PSD95 and PSD95 Protein Expression

**Figure 6:**
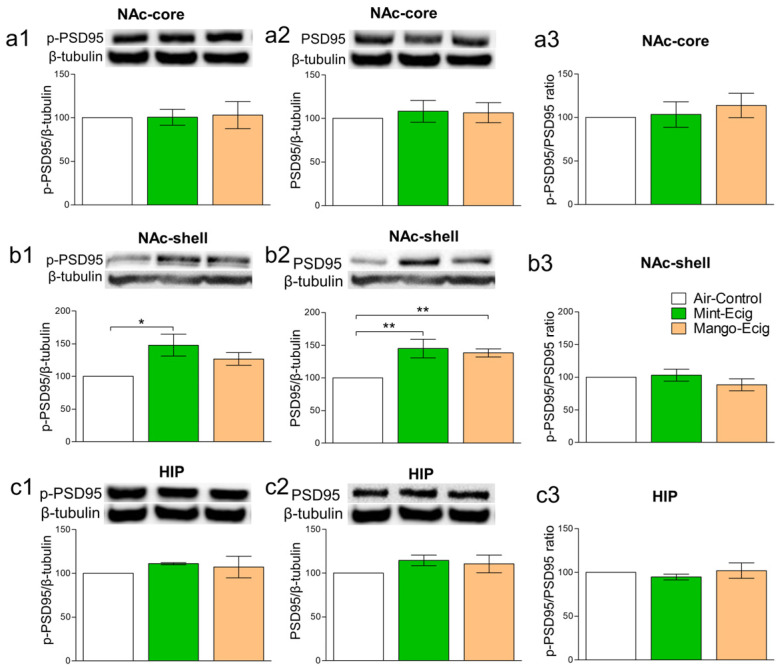
Effect of 3-month JUUL pods exposure on *p*-PSD95 and PSD95 protein expression. There was no significant change in *p*-PSD95 expression, (**a1**) and PSD95 expression, (**a2**) in the NAc-core. Accordingly, there was no significant change in the *p*-PSD95/PSD95 ratio in the NAc-core (**a3**). *p*-PSD95 expression was significantly increased in the e-cig mint group as compared to the air control (*p* < 0.01) and e-cig mango (*p* < 0.05) groups in the NAc-shell; (**b1**). PSD95 expression was significantly upregulated in e-cig mint (*p* < 0.05) and e-cig mango (*p* < 0.05) groups as compared to the air control; (**b2**). However, there was no significant difference in the *p*-PSD95/PSD95 ratio between all groups in the NAc-shell (**b3**). There was no significant change in the expression of *p*-PSD95, (**c1**) and PSD95, (**c2**). There was no significant change in the *p*-PSD95/PSD95 ratio in the HIP (**c3**). Data expressed as mean ± SEM, * *p* < 0.05 and ** *p* < 0.01, detected bands at ≈95 kDa for *p*-PSD95 and PSD95; *n* = 5–6/group. Abbreviations: NAc-core, nucleus accumbens core; NAc-shell, nucleus accumbens shell; HIP, hippocampus.

Previous studies exhibited the association between the postsynaptic scaffolding proteins such as PSD95 and the function of glutamate receptors [48,49,50,51,52]. We investigated whether changes in glutamate receptors/transporters, due to three-month JUUL exposure, were associated with PSD95 alteration. Three-month exposure to JUUL selectively increased the expression of *p*-PSD95 and PSD95 protein levels in the NAc-shell but not in the NAc-core and HIP. Data analysis showed that JUUL exposure for three months induced upregulation of *p*-PSD95 in the e-cig mint group (F_2,14_ = 5.31, *p* = 0.019; *n* = 5–6/group) and PSD95 in the e-cig mint and e-cig mango groups (F_2,14_ = 9.07, *p* = 0.003; *n* = 5–6/group) compared to the air control group in the NAc-shell. Three-month exposure to the e-cig mint group increased *p*-PSD95 protein compared to the air control group (*p* < 0.01), with no significant change when compared to the e-cig mango group. However, no significant change was observed between the air control and e-cig mango groups (Figure 6b1). Both e-cig mint and e-cig mango groups showed increases in PSD95 protein level compared to the air control group (*p* < 0.01, Figure 6b2). The ratio of *p*-PSD95 to PSD95 was not altered between all tested groups in the NAc-shell (Figure 6b3). Three-month exposure to JUUL did not alter the levels of *p*-PSD95 in the NAc-core (F_2,14_ = 0.02, *p* > 0.05; *n* = 5–6/group) and HIP (F_2,14_ = 0.52, *p* > 0.05; *n* = 5–6/group). Similarly, three-month exposure to JUUL did not alter the levels of PSD95 in the NAc-core (F_2,12_ = 0.19, *p* > 0.05; *n* = 5/group) and HIP (F_2,12_ = 1.17, *p* > 0.05; *n* = 5–6/group, Figure 6a1,a2,c1,c2). The ratio of *p*-PSD95 to PSD95 was not altered between all tested groups in the NAc-core and HIP (Figure 6b3,c3). The ratio was calculated by dividing the *p*-PSD95 expression level by the PSD95 expression level in the same blot.

## 4. Discussion

We demonstrated in this study that three-month exposure to mint- or mango-flavored JUUL pods (containing 59 mg /mL nicotine) altered the protein expression of mGluR1, mGluR5 and GLT-1 in the NAc-shell and HIP. The NAc is implicated in the dependence on drugs of abuse, including nicotine [53,54,55]. The NAc-core and NAc-shell received distinct but overlapping projections from different brain regions, including the HIP (for review see [56,57]), which trigger different functional responses. For example, the firing of dopaminergic projections from the VTA to NAc-shell is associated with the rewarding stimulus of the drug. In contrast to the NAc-shell, these projections sensitize the NAc-core and promote the transition to dependence status upon repeated exposure to drugs of abuse such as nicotine (for review see [58]). The NAc-shell receives glutamatergic projections from the infralimbic medial prefrontal cortex and the firing of these projections act to inhibit motivated reward-seeking behaviors [59,60]. Ample evidence demonstrated the role of excitatory neurotransmitters, such as glutamate, in mediating the reinforcing effects of nicotine in the NAc. Nicotine self-administration enhanced glutamate transmission in the NAc-shell, and this effect was attenuated by systemic treatment with mGluR2/3 agonist LY379268 [61]. Consistent with enhanced glutamate release, nicotine exposure increased the ratio of AMPA/NMDA excitatory postsynaptic currents, leading to higher excitability in dopaminergic neurons [62]. Moreover, microinfusions of selective mGluR5 antagonist 2-methyl-6-(phenylethynyl) pyridine (MPEP) into the NAc-shell attenuated nicotine self-administration in Wistar rats [63]. While mGluR5 expression was not altered in the NAc-core, our results showed that JUUL exposure induced downregulation of mGluR5 in the NAc-shell. Although both JUUL mint and mango flavors induced a similar change in mGluR5 expression, it seems that the mint flavor has a stronger mGluR5 downregulatory effect as revealed by the statistical analysis. Multiple mechanisms might be proposed to underlie the nicotine-seeking behavior. One hypothesis is that dependence on nicotine may depend on the action of glutamate at mGluR5 [35]. Interestingly, activation of mGluR5 exerts anti-inflammatory effects by inhibition of microglial activation and reduction in nitric oxide (NO), tumor necrosis factor-alpha (TNF-α) and reactive oxygen species (ROS) production in vitro [64], as well as reduction in the protein and mRNA expression of proinflammatory cytokines such as interleukin 1-beta (IL-1b), interleukin 6 (IL6) and TNF-α in vivo [65]. Previously, our laboratory showed that chronic ethanol consumption induced dysregulation of glucocorticoid receptors and upregulation of inflammatory mediators such as TNF-α, High mobility group box 1 protein (HMGB1) and receptor for advanced glycation end products (RAGE) in the NAc-shell but not in NAc-core [66,67], suggesting the disruption of NAc-shell activity in response to ethanol dependence. The decrease in mGluR5 expression in the NAc-shell upon three-month JUUL exposure may suggest disruption of glutamatergic activity in this brain area. Alternatively, the upregulation of mGluR1 expression in the NAc-shell further supports the dysregulation of glutamate homeostasis. mGluR1 is expressed in the brain areas of mesocorticolimbic pathways [68,69], and is involved in nicotine dependence. It has been shown that repeated administration of nicotine for three days increased mGluR1 expression in the amygdala and VTA [36]. Moreover, mGluR1 antagonist significantly attenuated nicotine-seeking behavior in a rat model [38]. Previously, our lab showed that rats consuming nicotine for four weeks exhibited upregulation of mGluR1 expression in the NAc [24]. Thus, JUULs may exert similar effects on mGluR1 expression to nicotine consumption.

Three-month JUUL exposure induced brain region-specific effects on the expression of GLT-1, i.e., downregulation of this protein in the NAc-shell but not in the NAc-core. This finding is in line with our previous study that showed that six-month exposure to e-cig vapor containing 24 mg/mL nicotine reduced the expression of GLT-1 in the striatum with no effects in the prefrontal cortex and HIP [17]. Moreover, chronic consumption of nicotine reduced GLT-1 expression in the NAc of P rats [24], and Wistar rats after 12 h of withdrawal from nicotine self-administration [70]. It has been suggested that reduction in GLT-1 expression in the NAc is linked to dependence on nicotine, ethanol, methamphetamine and cocaine [21,22,23,24]. Interestingly, xCT protein expression was not altered in the NAc and HIP after three-month JUUL exposure. It important to note that the effect of nicotine on the expression of xCT may depend upon the dose and length of exposure as well as the target brain region. For example, intravenous self-administration of nicotine (0.03 mg/kg/infusion) for 21 days reduced xCT expression in the NAc and VTA but not in the cortex and amygdala [70]. Moreover, chronic exposure to e-cig vapor containing 24 mg/mL nicotine was able to reduce xCT expression in the striatum and HIP [17]. Knowing that neuroadaptation to the effects of nicotine may be developed upon repeated exposure (for review see [71]), we postulated here that JUUL pods containing a high nicotine concentration may be associated with a neuroadaptive mechanism involving the expression of xCT in the NAc and HIP. Together, these findings suggest dysregulation in the glutamatergic system in the NAc-shell, the brain region involved in the inhibition of drug-seeking behavior, but not the NAc-core, which is associated with reinstatement to drugs of abuse-seeking behaviors [72].

JUUL-induced alterations in the NAc-shell were accompanied with increased expression of phosphorylated and total scaffolding protein PSD95. The highly abundant postsynaptic PSD95 regulates synaptic plasticity and neurotransmission, especially AMPA and NMDA glutamatergic neurotransmission [73,74,75,76]. Acute nicotine treatment in mice increased PSD95, which in turn increased the availability of AMPA receptors [77]. In accordance, chronic nicotine exposure increased the expression of PSD95 and AMPA receptors in the midbrain of a mouse model [78]. Our results may indicate the presence of higher synaptic plasticity in the NAc-shell. This is supported by the fact that chronic nicotine self-administration was associated with altered expression of ionotropic receptors [79], which contribute to the hyperglutamatergic state [80] as well as increased neuronal plasticity [81].

Three-month JUUL exposure can lead to differential effects on the protein expression of mGluR5 and GLT-1 in the NAc and HIP. Three-month JUUL exposure significantly increased mGluR5 and GLT-1 expression in the HIP. The HIP has a major role in mediating the rewarding effect of nicotine and associated learning and memory during nicotine dependence (for review see [82]). Acute and chronic nicotine administration enhanced learning and memory, and these effects were associated with enhanced hippocampal long-term potentiation (LTP) [83,84,85]. Alternatively, chronic nicotine exposure was associated with neuroadaptation, which was associated with the development of tolerance to the acute and withdrawal effects of nicotine [86,87]. Enhancement of LTP was associated with activation of mGluR5 in male rats exposed to nicotine for two weeks [84]. Our findings in the HIP suggest that three-month exposure to JUUL pods containing high nicotine levels may lead to neuroadaptation, i.e., increased mGluR5 and GLT-1 expression. However, mint-flavored JUUL pods were associated with relatively higher mGluR5 and GLT-1 expression compared to mango-flavored JUUL pods. Future studies are warranted to address the differential effects of different additive flavors on the addictive properties of e-cigs. One limitation in our study is that we tested female mice only. It is recommended in future studies to test the effects of JUUL pods on male mice to determine whether there are sex differences. Future studies may be extended to investigate the expression of current protein markers in other brain regions such as the VTA to reveal the region-specific effects after JUUL exposure. Lastly, further studies are warranted to investigate the effects of e-cigs in target proteins using immunostainings.

In summary, this study demonstrated that three-month exposure to e-cig vapor, containing a high nicotine concentration, induced differential effects on several targets of the glutamatergic system in the NAc subregions and HIP, and probably similar to the effects induced by chronic nicotine exposure. Three-month JUUL exposure induced an increase in mGluR1 expression and reduction in mGluR5 and GLT-1 expression in the NAc-shell. However, the expression of mGluR5 and GLT-1 were increased in the HIP, with no change in their expression in the NAc-core. These findings suggest dysregulation of glutamatergic system activity in the NAc-shell subregions and HIP in response to chronic e-cig exposure.
